# Highly Diverse Efficacy of Salvage Treatment Regimens for Relapsed or Refractory Peripheral T-Cell Lymphoma: A Systematic Review

**DOI:** 10.1371/journal.pone.0161811

**Published:** 2016-10-06

**Authors:** Ya-Ting Yang, Cheng-Jeng Tai, Chiehfeng Chen, Hong-Cheng Wu, Natalia Mikhaylichenko, Hsien-Tsai Chiu, Yun-Yi Chen, Yi-Hsin Elsa Hsu

**Affiliations:** 1 Institute of Health Policy and Management, National Taiwan University, 17 Xu-Zhou Rd., Taipei, 100, Taiwan; 2 Golden Dream Think Tank and Research Center, 17 Songjiang Rd., Taipei, 104, Taiwan; 3 School of Health Care Administration, Taipei Medical University, 250 Wuxing Street, Taipei, Taiwan; 4 Division of Hematology and Oncology, Department of Internal Medicine, Taipei Medical University Hospital, 252 Wuxing Street, Taipei, 110, Taiwan; 5 Department of Internal Medicine, School of Medicine, College of Medicine, Taipei Medical University, 250 Wuxing Street, Taipei, 110, Taiwan; 6 Center for Evidence-Based Medicine, Taipei Medical University, 250 Wuxing Street, Taipei, 110, Taiwan; 7 Department of Public Health, School of Medicine, College of Medicine, Taipei Medical University, 250 Wuxing Street, Taipei, 110, Taiwan; 8 Division of Plastic Surgery, Department of Surgery, Wan Fang Hospital, Taipei Medical University, NO.111, Section 3, Hsing-Long Rd, Taipei, 116, Taiwan; 9 Evidence-Based Medicine Center, Wan Fang Hospital, Taipei Medical University, NO.111, Section 3, Hsing-Long Rd, Taipei, 116, Taiwan; 10 Nevron, International Medical Center, Vladivostok, Russia; 11 Department of Public Health, China Medical University, 91 Hsueh-Shih Road, Taichung, 404, Taiwan; Shanghai Jiao Tong University School of Medicine, CHINA

## Abstract

**Background:**

The goal of this study was to perform a systematic review to examine the efficacy and safety of various salvage therapy regimens on patients with relapsed/refractory PTCL.

**Method:**

The electronic searches were performed using PubMed, Cochrane Library, EMBASE, and Web of Science from inception through June 2015, with search terms related to relapsed/refractory PTCL, salvage chemotherapy regimens, and clinical trials. An eligible study met the following inclusion criteria: (1) Patients had refractory or relapsed PTCL; (2) drug regimens were used for salvage therapy; (3) the study was a clinical trial; (4) the study reported on a series of at least 10 patients of PTCL.

**Results:**

Of 35 records identified, a total of 14 studies were eligible for systematic reviews, and 12 different salvage regimens were investigated. A total of 618 relapsed/refractory PTCL patients were identified. The ORRs ranged from 22% for those treated with lenalidomide to 86% for those with brentuximab vedotin. By the three most frequent subtypes, the ORRs ranged from 14.2% to 71.5% for patients with the PTCL-NOS subtype, 8% to 54% for AITL subtypes, and 24% to 86% for the ALCL subtype. The medians of DOR, PFS, and OS ranged from 2.5 to 16.6 months, 2.6 to 13.3 months, and 3.6 to 14.5 months, respectively. The most frequently reported grade 3 or 4 adverse events (AEs) were hematological AEs, such as neutropenia and thrombocytopenia.

**Conclusion:**

The efficacy of salvage therapy regimens is highly diverse for patients with relapsed/refractory PTCL; this heterogeneity in therapeutic effects might be due to the diversity in mechanisms, PTCL subtype distribution, and/or numbers/profiles of prior therapy. Comparative studies with matched pair analysis are warranted for more evidence of the salvage treatment effect on relapsed or heavily pretreated patients with PTCL.

## Introduction

Peripheral T-cell lymphomas (PTCL) are a group of relatively rare, clinically and biologically heterogeneous lymphoproliferative disorders that develop in mature blood cell called “T cells” and “natural killer (NK) cells [1], which account for 10–15% of all non-Hodgkin’s lymphomas (NHL) in the Western population [2]. Generally, PTCLs are significantly rarer and more difficult to treat when compared to their B-cell counterparts [3], either due to the paucity of large trials carrying the evidence to suggest specific therapeutic approaches or due to the biology of the disease. The 2008 World Health Organization classification system contains 22 different T-cell lymphoma subgroups, which are distinct regarding pathology, clinical presentation, response to therapy, and expression of surface markers [2,4,5]. According to the International T-Cell Lymphoma Project [6], the most common subtypes are PTCL-not otherwise specified (PTCL-NOS) (25.9%), angioimmunoblastic T-cell lymphoma (AITL) (18.5%), and anaplastic large cell lymphoma (ALCL) (12.1%), which might be positive or negative for anaplastic lymphoma kinase (ALK). However, the distributions of the various lymphoma subtypes are geographically diverse; for example, PTCL-NOS is the most common subtype in North American and Europe, while adult T-cell leukemia/lymphoma (ATLL) is most common in Asia [6].

Patients with PTCL are characterized by poor treatment outcomes with conventional chemotherapy and no established standards of care for patients with relapsed and refractory settings [7]. Standard first-line therapy consists of CHOP (cyclophosphamide, doxorubicin, vincristine, and prednisone) or a CHOP-like regimen. Nevertheless, therapeutic responses to this approach have been neither appropriate nor durable [8]. According to the International T-Cell Lymphoma Project, the 5-year overall survival rate is poor for most subtypes: 32% for PTCL-NOS and AITL, and 14% for ATLL [6]. With conventional chemotherapy alone, a population-based cancer registry study showed that median overall survival (OS) is only 6.5 months for patients after first relapse or progression of PTCL [9]. The generally poor outcomes observed in PTCL patients emphasize the urgent need for alternative therapy [10]. Moreover, due to its rarity and the heterogeneity of subtypes, randomized controlled trials comparing different treatment approaches for PTCL are very limited [5]. Several novel approaches have been evaluated in single-arm phase I and II studies, mainly in patients with relapsed/refractory disease who had particularly poor prognoses [5].

In the last 5 years, several therapeutic agents with novel mechanisms of action have been approved by the US Food and Drug Administration (FDA) for patients with relapsed/refractory PTCL [11,12], including pralatrexate [13], romidespsin [14], brentuximab vedotin [15], and belinostat [16]. According to the National Comprehensive Cancer Network (NCCN) guidelines [17], FDA-approved agents, promising single agents, and some combination chemotherapies were advocated as the second-line therapy for relapse/refractory PTCL, both for candidates and non-candidates for stem cell transplantation (SCT). To the extent of our knowledge, there are no systematic reviews for the salvage treatment regimens focused on relapsed/refractory PTCL, as only narrative reviews exist [5,7]. In order to capture the changing landscape of the salvage treatment for relapsed/refractory PTCL, we conducted a systematic review regarding the efficacy and safety of salvage treatment regimens among patients with relapsed/refractory PTCL.

## Methods

### Literature Search Strategy

According to NCCN guidelines (Version 5, 2014) [17] for PTCL, the suggested treatment regimens of second-line therapy are as follows (in alphabet order): (1) single-agent therapy: alemtuzumab, belinostat, bortezomib, brentuximab vedotin, cyclosporine, gemcitabine, pralatrexate, romidepsin; (2) combination therapy: DHAP (dexamethasone, cytarabine, cisplatin), dose-adjusted EPOCH (etoposide, prednisolone, vincristine, cyclophosphamide, doxorubicin), ESHAP (etoposide, methylprednisolone, cytarabine, cisplatin), GDP (gemcitabine, dexamethasone, cisplatin), GemOx (gemcitabine, oxaliplatin), ICE (ifosfamide, carboplatin, etoposide), MINE (mesna, ifosfamide, mitoxantrone, etoposide). The electronic searches were performed using PubMed, Cochrane Library, EMBASE and Web of Science from their date of inception to June 12, 2015. To identify specific and relevant studies, we used a search strategy based on patient population (relapsed/refractory PTCL), treatments (NCCN guide suggested regimens and other salvage chemotherapy), and study designs (phase II clinical trials) (Tables A and B in S1 File). The keywords in the extensive electronic literature search, such as “Peripheral T-cell Lymphoma,” “Relapsed or Refractory,” “Salvage therapy,” and “Clinical Trial,” were transformed into MeSH terms. The references from the NCCN guidelines for relapsed/refractory PTCL were also accessed for eligibility in the review process (as another resource). All identified articles were systematically assessed using the inclusion and exclusion criteria.

### Selection Criteria

In this systematic review, an eligible study met the following inclusion criteria: (1) Patients had refractory or relapsed PTCL; (2) Drug regimens were used for salvage therapy or relevant to those previously defined; (3) the study was a clinical trial (phase II); (4) the study reported on a series of at least 10 patients of PTCL to prevent bias arising from small sample populations.

Exclusion criteria were as follows: (1) Patients were irrelevant to any subtype of PTCL (cutaneous T-cell lymphoma [CTCL] are excluded) or were not treated previously (not relapsed or refractory); (2) Drug regimen was used as front therapy, not salvage therapy; (3) Retrospective studies, phase I clinical trial, review paper, comment, letter, abstract, editorial; (4) Patient number of PTCL was less than 10; (5) Absence of efficacy outcome such as overall response rate (ORR).

Rather than conduct a narrow search for specific chemotherapy combinations, we separately searched each single agent within a given combination in order to generate the broadest possible search results that would include all possible treatment combinations in which that agent appeared. For example, dexamethasone, a common agent in chemotherapy, was searched alone so that both DHAP and GDP appeared as possible combinations. However, if only a single agent was found alone in some studies, these papers were also included in the systematic review process for eligibility.

### Quality Assessment

The common quality assessment tools suggested for randomized controlled trials (RCTs) were “Cochrane’s risk of bias checklist” and the “SIGN50 RCT checklist”, and another tool for non-randomized studies of interventions was also recommended [18]. However, since the studies we included were more likely single-armed, these three tools might not have been able to adequately evaluate the study quality of these selected papers. Here, we proposed a modified 18-item checklist that was often used in quality appraisals of case-series or one-group studies [19]. The minimum number of satisfied checklist items necessary for a study to be classified as high quality was determined to be 10.

### Data extraction and critical appraisal

The primary outcome measure was ORR, and secondary outcome measures included duration of response (DOR), progression-free survival (PFS), and OS. The median of these durations (including range or 95% confidence interval [CI]) was extracted. With regard to safety outcomes, World Health Organization (WHO) grade 3 or 4 hematological and non-hematological adverse events (AEs) were extracted respectively. Moreover, extracted data also included type of study, PTCL subtype, prior therapy (number of prior therapies, autologous hematopoietic stem cell transplantation [auto-HSCT]), demographic characteristics of patients (age, gender, and ethnics), and median follow-up time. Quality-control procedures for the data extraction comprised of verification of all extracted data with their original sources by a second researcher. The study selection, quality assessment, and data extraction were performed independently by two reviewers using a standardized form. The discrepancies between the two reviewers were resolved through discussion and consensus.

### Data Analysis

A narrative review of these studies was presented. Data on characteristics of studies were presented, if available. The ORR and 95% CI across various studies were graphically presented, and the corresponding plot was conducted by Comprehensive Meta-Analysis, Version 2 (Biostat Inc., Englewood, NJ, USA).

## Results

### Screening process and quality of included studies

A total of 35 records were selected for abstract screening (databases = 17, other resources = 21, duplicate records = 3). A total of 21 records (databases = 16; other resources = 5) were identified for full-text screening. A total of 14 records were excluded either because only their abstracts were available (n = 3), the patients did not pertain to any subtype of PTCL or relapsed/refractory setting (n = 6), or the therapies (n = 3) or study designs (n = 2) were irrelevant. Out of the 21 records, 7 records were further excluded due to the sparse number of PTCL patients (number of PTCL patients < 10, n = 5), study design (retrospective study, n = 1), and a trial with duplicate population (n = 1).

**Fig 1:**
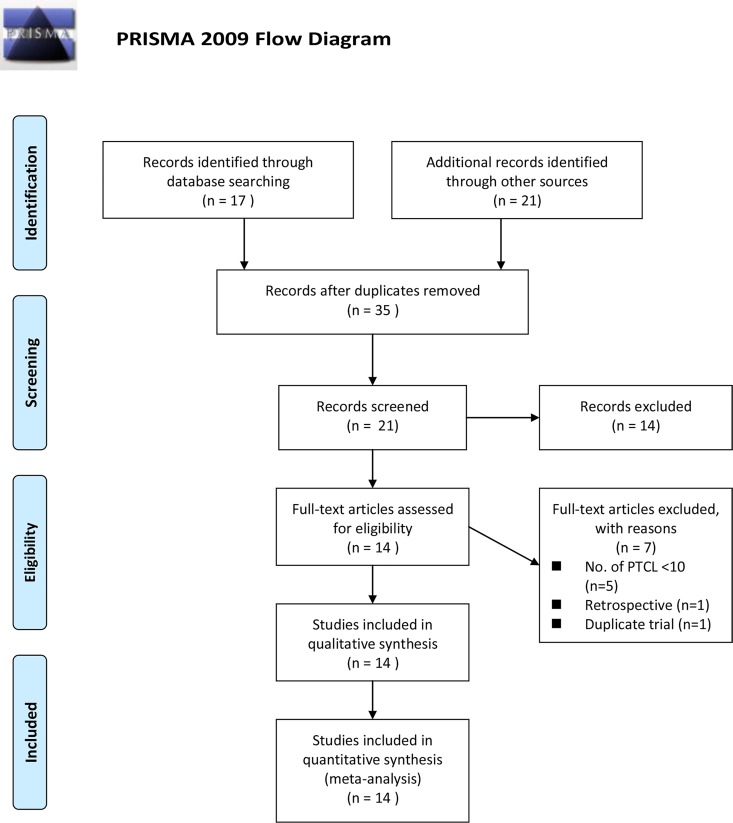
PRISMA 2009 Flow Diagram.

The screening process is shown as a PRISMA flow chart (Fig 1). Using a modified 18-item checklist to evaluate study quality, all 14 studies that satisfied a minimum of 10 or more checklist items were considered high quality, with the number of items satisfied by each study ranging from 10 to 17. The detail records of quality assessment are shown in the supplementary materials (Table C in S1 File).

### Study characteristics and Primary outcome

The basic characteristics of the selected studies are shown in Table 1. A total of 882 patients with NHL were initially identified from the 14 studies, and 618 of them were pertaining to relapsed/refractory PTCL. The most common subtypes were PTCL-NOS (n = 303, 49.0%), ALCL (n = 128, 20.7%), and AITL (n = 123, 19.9%). The patient numbers of relapsed or refractory PTCL ranged from 14 to 130. A total of 12 regimens were investigated, including 4 US FDA approved novel single-agent regimens (pralatrexate [13], romidespsin [14], brentuximab vedotin [15,20], belinostat [16]), 5 promising single-agent regimens (alemtuzumab [21], bendamustine [22], gemcitabine [23,24], lenalidomide [25], zanolimumab [26]), and 3 combination chemotherapies (13-cRA+interferon-α [27], A-DHAP [28], ICE [29]). The median age of study population from the included studies ranged from 46 to 66 years, and the median number of prior systematic therapies ranged from 1 to 3. Five of the 14 studies reported that some patients were previously treated with auto-HSCT, with the percentages ranging from 9% to 26%. Regarding clinical response assessments, only 4 of the 14 studies conducted independent or central reviews that indicated more rigorous assessment criteria than only local investigator (Table 1).

**Table 1:** Summary of characteristics of selected studies for patients with relapsed or refractory peripheral T-cell lymphoma treated with various salvage therapies.

Study (year)	Regimen	Mechanism	No. of Patients, All/RR-PTCL	Age (year), median (range)	No. of prior therapy, median (range)	Prior auto-HSCT (%)	Independent Central Review
Enblad et al. (2004)	Alemtuzumab	Anti-CD52 monoclonal antibody	14/14	61 (53–79)	2 (1–4)	NR	No
Foss et al. (2015)	Belinostat	HDAC inhibitor	53/24	64 (22.8–76.3)	N.R.	5 (20.8%)	No
Damaj et al. (2013)	Bendamustine	Alkylating agent	60/58	66 (43–87)	1 (1–3)	NR	No
Pro et al. (2012)	Brentuximab vedotin	Anti-body drug conjugate	58/58	52 (14–76)	2 (1–6)	15 (26%)	Yes
Horwitz et al. (2014)	Brentuximab vedotin	Anti-body drug conjugate	35/35	64 (33–83)	2 (1–9)	3 (9%)	No
Zinzani et al. (2000)	Gemcitabine	Nucleoside analog	44/14	58 (25–82)*	3 (2–5)*	NR	No
Zinzani et al. (2010)	Gemcitabine	Nucleoside analog	39/20	54 (32–78)*	3 (2–8)*	NR	No
Morschhauser et al. (2013)	Lenalidomide	Immunomodulatory	54/51	64.5 (39–86)*	3 (1–11)*	NR	No
O'Connor et al. (2011)	Pralatrexate	Antifolates	111/109	57.5 (21–85)	3 (1–13)	18 (16%)	Yes
Coiffier et al. (2012)	Romidepsin	HDAC inhibitor	130/130	61 (20–83)	2 (1–8)	21 (16%)	Yes
d’Amore et al. (2010)	Zanolimumab	Anti-CD4 monoclonal antibody	21/21	69 (26–85)	2 (1–5)	NR	No
Huang et al. (2002)	13-cRA+interferon-α	Combination therapy	17/17	47 (18–77)	1 (1–3)	NR	Yes
Seok et al. (2012)	A-DHAP	Combination therapy	24/24	49 (23–60)	NR	NR	No
Zelenetz et al. (2003)	ICE	Combination therapy	222/43	46 (NR)*	NR	No	No

*indicates the statistics including data for some subtype other than PTCL

Abbreviations: A-DHAP: alemtuzumab, dexamethasone, cytarabine, cisplatin; ICE: ifosfamide, carboplatin, etoposide; HDAC: histone deacetylase; PTCL: peripheral T-cell Lymphoma; RR: relapsed or refractory; PTCL-NOS: peripheral T-cell Lymphoma, not otherwise specified; ALCL: anaplastic large cell lymphoma, ALCL(+): anaplastic lymphoma kinase positive ALCL, ALCL(-):anaplastic lymphoma kinase negative ALCL; AITL: angioimmunoblastic T-cell lymphoma; ENKTCL: extra-nodal NK/T cell lymphoma, nasal type; auto-HSCT: autologous hematopoietic stem cell transplantation; ORR: overall response rate; NR: not reported.

### Primary outcome: overall response rate

The efficacy outcomes of relapsed/refractory PTCL treated with salvage therapy are shown in Table 2. Regarding primary efficacy outcome, the ORRs of included studies ranged from 22% treated with lenalidomide to 86% with brentuximab vedotin. Divided by the most frequently presented subtypes, the ORRs ranged from 14.2% to 71.5% for patients with the PTCL-NOS subtype, 8% to 54% for AITL subtypes, and 24% to 86% for the ALCL subtype, respectively (Table 2).

**Table 2:** Summary of characteristics of selected studies for primary and secondary outcomes in patients with relapsed or refractory peripheral T-cell lymphoma treated with various salvage therapies.

		Primary outcome: ORR (%) (95% CI)				Secondary outcomes, median (95% CI)		
Study (year)	PTCL subgroup	All PTCL subtypes	PTCL-NOS	AITL	ALCL	DOR (month)	PFS (month)	OS (month)
Enblad et al. (2004)	PTCL-NOS 10	36	50	NR	NR	2.5 (1–8)	NR	NR
Foss et al. (2015)	PTCL-NOS 13, ALCL 3, AITL 3	25 (7.7–39.1)	NR	NR	NR	3.63 (0.23–15.3)	2.73 (1.2-NE)	NR
Damaj et al. (2013)	AITL 32, PTCL-NOS 23, ALCL 2	50	NR	NR	NR	3.5 (1–20.7)	3.63 (2.41–5.19)	6.27 (5.12–9.59)
Pro et al. (2012)	ALCL(+) 16, ALCL(-) 42	86 (74.6–93.9)	NR	NR	All: 86 (74.6–93.9), ALCL(-): 88, ALCL(+): 81	12.6 (5.7-NE)	13.3 (6.9-NE)	NE (14.6-NE)
Horwitz et al. (2014)	PTCL-NOS 22, AITL 13	41 (24.6–59.3)	33 (14.6–57)	54 (25.1–80.8)	NR	7.6 (1.3–14+)	2.6	NR
Zinzani et al. (2000)	PTCL-NOS 14	71.5	71.5	NR	NR	CR: 15 (6–22), PR: 10 (2–15)	NR	NR
Zinzani et al. (2010)	PTCL-NOS 20	55	55	NR	NR	CR: 34 (15–120)	NR	NR
Morschhauser et al. (2013)	AITL 26, PTCL-NOS 20	22*	20	31	NR	AITL, CR: 3.6 (3.5-NE)	All: 2.5 (1.8–4.6), AITL: 4.6 (1.8–8.2), non-AITL: 1.9 (1.6–3.3)	NR
O'Connor et al. (2011)	PTCL-NOS 59, ALCL 17, AITL 13	29*	32 (21–46)	8 (0–36)	35 (14–62)	10.1 (3.4-NE)	3.5 (1.7–4.8)	14.5 (1–24.1)
Coiffier et al. (2012)	PTCL-NOS 69, AITL 27, ALCL(-) 21	25	29	30	ALCL(-): 24	16.6 (<0.1–34)	4	NR
d’Amore et al. (2010)	AITL 9, PTCL-NOS 7, ALCL 4	24 (8, 47)	14.3	33.3	25.0	NR	NR	NR
Huang et al. (2002)	PTCL-NOS 7, Ki-1 ALCL 6	31.3 (5.7–56.8)	14.2	NR	Ki-1 ALCL: 66.6	2.5	2.7	3.6
Seok et al. (2012)	PTCL-NOS 13, ENKTCL 8	50	69.2	NR	NR	2.93 (0.93–4.93)	NR	6 (4.2–7.8)
Zelenetz et al. (2003)	PTCL-NOS 26, ALCL 17	62.7	54	NR	77	NR	NR	NR

*indicates the statistics including data for some subtype other than PTCL

Abbreviations: A-DHAP: alemtuzumab, dexamethasone, cytarabine, cisplatin; ICE: ifosfamide, carboplatin, etoposide; HDAC: histone deacetylase; PTCL: peripheral T-cell Lymphoma; RR: relapsed or refractory; PTCL-NOS: peripheral T-cell Lymphoma, not otherwise specified; ALCL: anaplastic large cell lymphoma, ALCL(+): anaplastic lymphoma kinase positive ALCL, ALCL(-):anaplastic lymphoma kinase negative ALCL; AITL: angioimmunoblastic T-cell lymphoma; ENKTCL: extra-nodal NK/T cell lymphoma, nasal type; auto-HSCT: autologous hematopoietic stem cell transplantation; ORR: overall response rate; CR: complete response: PR: partial response; NR: not reported; NE: non-estimable DOR: duration of response; PFS: progression-free survival; OS: overall survival.

### Secondary outcomes: duration of response, progression-free survival, overall survival

Nine of the 14 included studies reported DOR, and the medians ranged from 2.5 to 16.6 months. Seven of the 14 included studies reported PFS, and the medians ranged from 2.6 to 13.3 months. Only 5 of the 14 included studies reported OS, and the medians ranged from 3.6 to 14.5 months (Table 2).

### Safety outcome: WHO grade 3 or 4 adverse events

The most frequent WHO grade 3 or 4 AEs for patients with relapsed or refractory PTCL treated by salvage therapy are summarized in Table 3. In general, grade 3 or 4 AEs were more likely hematological AEs for patients with relapsed or refractory PTCL, such as neutropenia (14% to 56%), thrombocytopenia (12% to 38%), leukopenia (28.5% to 78.2%), and anemia (11% to 18%). Those treated with gemcitabine [23, 24] did not suffer from grade 3 or 4 hematological AEs. The most frequently reported non-hematological AEs were infection (15% to 23%). Moreover, those treated with brentuximab vedotin reported grade 3 or 4 peripheral sensory neuropathy (9% to 12%) [15, 20], while those treated with pralatrexate showed grade 3 or 4 mucositis (22%) [13].

**Table 3:** Summary of most frequent adverse events with WHO grade 3 or 4 for patients with relapsed or refractory peripheral T-cell lymphoma treated by various salvage therapy.

Study (year)	Regimen	No. of Patients treated	Hematological AE (%)							Non-hematological AE (%)
			leukopenia	neutropenia:		thrombocytopenia:	lymphopenia:	anemia:	hyperkalemia:	
Enblad et al. (2004)	Alemtuzumab	14	28.5		21.4	14.2				NR
Foss et al. (2015)	Belinostat	24					62.5			NR
Damaj et al. (2013)	Bendamustine	60			56	38				infection: 20
Pro et al. (2012)	Brentuximab vedotin	58			21	14				peripheral sensory neuropathy: 12
Horwitz et al. (2014)	Brentuximab vedotin	35			14				9	peripheral sensory neuropathy: 9
Zinzani et al. (2000)	Gemcitabine	44*	No grade 3 and grade 4 hematologic toxicity							NR
Zinzani et al. (2010)	Gemcitabine	39*	No grade 3 and grade 4 hematologic toxicity							NR
Morschhauser et al. (2013)	Lenalidomide	54			15	20				gastrointestinal disorder: 17, infection: 15
O'Connor et al. (2011)	Pralatrexate	111*			15	32		18		mucositis: 22
Coiffier et al. (2012)	Romidepsin	131			20	24		11		infection: 19
d’Amore et al. (2010)	Zanolimumab	21	NR							NR
Huang et al. (2002)	13-cRA+interferon-α	17				12		12		infection: 23.5, fever: 12, fatigue: 12
Seok et al. (2012)	A-DHAP	24	79.2			16.7				NR
Zelenetz et al. (2003)	ICE	222*	NR							NR

*indicates the statistics including data for some subtype other than PTCL

Abbreviations: A-DHAP: alemtuzumab, AEs, adverse events; dexamethasone, cytarabine, cisplatin; ICE: ifosfamide, carboplatin, etoposide; HDAC: histone deacetylase; PTCL: peripheral T-cell Lymphoma; RR: relapsed or refractory.

### Cross-evaluation by both relative efficacy and safety

**Fig 2:**
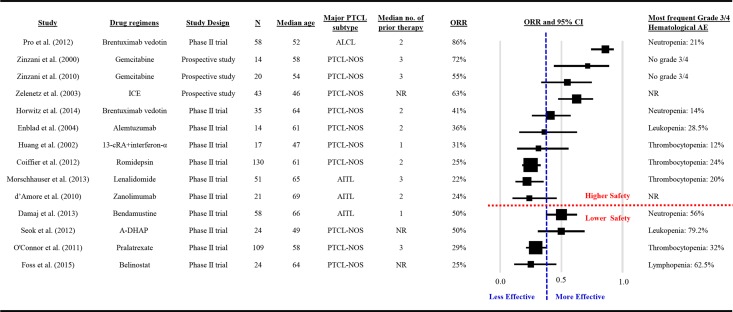
Summary ORR plots of studies.

An ideal therapy regimen should be both effective and well-tolerated. The summary plot of ORR of the 14 studies is shown in Fig 2. The vertical, blue, dashed line indicates the threshold of relative efficacy (based on median of ORRs across studies, ORR > 38%), and the horizontal, red, dashed line indicates the cutoff of relative safety (most frequent grade 3 or 4 hematological AEs < 30%). If only phase II clinical trials were considered, those treated by brentuximab vedotin seemed to show both relatively higher efficacy and relatively higher safety (Fig 2).

## Discussion

### Summary findings in this systematic review

In this study, we conducted a systematic review regarding salvage treatments on patients with relapsed or refractory PTCL. Systematic reviews can provide convincing and reliable evidence relevant to many aspects of medicine and health care; however, the conclusions are less clear when the included studies have differing results [30]. Through 2 levels of screening for eligible studies, a total of 14 studies covering 12 regimens were systematically reviewed. An apparent variation was observed in the ORRs across studies and regimens, along with various sample sizes, diverse distribution of PTCL subtypes, and different profiles of prior therapy. In this review, patients with the ALCL subtype responded to the salvage treatment (ORR: 24% to 86%), followed by those with PTCL-NOS (ORR: 14.2% to 71.5) and AITL (ORR: 8% to 54%). Among all 12 regimens, brentuximab vedotin had impressive ORR among those with the ALCL subtype (ORR: 86%). For the AITL subtype (ORR: 54%), romidepsin showed longer median duration of response (16.6 months) and pralatrexate showed longer median survival (14.5 months).

To the best of our knowledge, this is the first systematic review concerning salvage therapy regimens on relapsed/refractory PTCL. Our narrative review with comprehensive therapeutic options for relapsed or refractory PTCL, including different novel agents and stem cell transplantation, pointed out the development of new and effective treatment strategies for improving overall outcomes in patients with PTCL [5]. Moreover, a systematic review and meta-analysis was conducted for front-line anthracycline-based chemotherapy for PTCL, and it suggested that there is marked heterogeneity across PTCL subtypes in the benefits of anthracycline-based chemotherapy [8]. Compared to common aggressive B-cell lymphomas, patients with PTCL were more likely refractory to initial therapy and those with clinical responses tended to have shorter progression-free survival [1]. This indicates that novel agents and regimens are needed to improve outcomes for these patients, especially for the relapsed/refractory settings.

### Heterogeneity in treatment efficacy across different salvage regimens

In 1994, the REAL Classification system for NHL explicitly distinguished B-cell and T-cell lymphomas with new subtypes based upon contemporary morphological, immunological, and genetic techniques [31]. The treatment paradigms for aggressive B-cell lymphoma were not effective for T-cell lymphoma [7]. Some salvage treatments previously stated to be effective for general relapsed/refractory lymphomas, such as DHAP [32] and ESHAP [33], were not as effective when administrated in relapsed/refractory PTCL specifically, as the clinical prognoses and treatment responses of B- and T-cell lymphomas are quite different. Even within PTCL, the prognoses and responses to therapy were varied across subtypes. An international study showed that the highest 5-year OS rates were observed in patients with primary cutaneous ALCL (90%) and ALK+ ALCL (70%), while poor 5-year OS rates were found for PTCL-NOS and AITL (both 32%) [6]. In this systematic review, the 14 included studies were highly heterogeneous in distribution of PTCL subtypes. Table 1 displays the numbers and profiles of prior therapy, demographic characteristics, review approaches for outcome assessment, and primary and secondary outcomes. These factors might be confounders for outcomes of different regimens. There is no head-to-head comparison among these studies. Some studies included heterogeneous subtype, but some recruited only a single subtype. There were two different treatment strategies: one single regimen constantly effective for different subtypes [13, 14], or subtype-specific regimens for optimal response [15]. Future studies are warranted to demonstrate which approach is more beneficial to relapsed/refractory PTCL.

### Adverse events: well-tolerated or treatable?

One study did not report data of treatment toxicity [29], one did not show clear numbers of grade 3 or grade 4 adverse event [26], and two claimed no grade 3 or 4 hematologic toxicity [23, 24]. Ten studies had different frequencies of grade 3 or 4 hematologic AEs. An updated study on romidepsin demonstrated durable response with 28-month median DOR—the extended treatment and longer follow-up did not affect the reported safety profile of romidepsin. Regarding non-hematologic toxicity, 22% patients who received pralatrexate reported grade 3 or 4 mucositis. Generally, the adverse events caused by pralatrexate were manageable and most patients recovered and continued to receive the target dose of 30 mg/m^2^ pralatrexate for the duration of the therapy [12]. Leucovorin was demonstrated to reduce toxicity of pralatrexate without losing efficacy [34]. The adverse events were more severe if combination therapy was applied. The combination of single agents might result in improved outcomes; however, it also increases risk of treatment-related toxicity.

#### Therapeutic options

Outcomes of patients with PTCL are generally poor, with no well-established care standards for relapsed/refractory PTCL. Many options remain in the therapeutic abyss [7]. Treatment guidelines for PTCL [17,35] included stem cell transplantation as one therapeutic option. The fact that 5 of the 14 included studies included patients treated with auto-HSCT (9% to 20.8%) suggests that prior use of the therapy must have achieved enough of a response to warrant inclusion as a treatment option; however, the ultimate outcome of auto-HSCT in these 5 studies was neither durable nor efficacious. Allogeneic HSCT was demonstrated as a curative therapy for relapsed/refractory PTCL, provided that patients had suitable response to prior chemotherapy [36]. Studies showed the impressive efficacy of brentuximab vedotin in relapsed/refractory PTCL [15, 20]. The potential of immunotherapy was also promising for either front-line or second-line therapy for PTCL [35]. With many novel single agents demonstrating efficacy in relapsed/refractory PTCL, integrating these agents into conventional front-line chemotherapy is a major focus of study [12]. The phase III randomized trials comparing up-front multi-agent chemotherapy regimens and convention chemotherapy among previously untreated PTCL were reported ongoing [35].

Due to the rarity and heterogeneity of PTCL subtypes, the evidence for RCTs is very limited. By matched paired analysis [37,38], we could simulate randomized control data and compare patients treated with novel agents to those with conventional agents during a similar time period or with similar prognostic factors. So far, the largest cohort of PTCL consists of 1,314 cases from 22 centers worldwide [6]. The international, multicenter studies of large PTCL cohorts are valuable to establish a databank for long-term evaluations for patient outcomes and provide evidence-based practices [39].

### Limitation

Our report presented a systematic review of best available data from existing publications on the salvage treatment of relapsed/refractory PTCL. Even though 4 electronic databases were searched, the studies regarding salvage treatment for relapsed/refractory PTCL were still very limited. Since PTCL is a relatively rare group of NHL, those with relapsed or refractory disease are even fewer. Moreover, lack of randomized control trials constrained the pool estimate of treatment effect. While the limited number of trials suggested an urgent need for relapsed/refractory PTCL treatment, it also decreased our likelihood for publication bias. Past studies have suggested that systematic reviews of randomized trials are the best strategy for appraising evidence; however, there have been instances where the findings of meta-analyses were later contradicted by large trials [40].

## Conclusion

The efficacy of salvage therapy regimens is highly diverse for patients with relapsed/refractory PTCL, and the heterogeneity in the therapeutic effects might be due to the diversity in mechanisms, PTCL subtype distribution, numbers/profiles of prior therapy, and/or the assessment approaches. System review could provide convincing evidence relevant to many aspects of medicine; however, the conclusions are less clear when the included studies have differing effects, especially based on a limited number of small trials. The results of impressive efficacy and well-tolerated toxicity from these salvage therapy regimens should be treated with considerable caution. Use of comparative studies with matched pair analysis is warranted for more evidence on salvage treatment effects on relapsed or heavily pretreated patients with PTCL.

## Supporting Information

S1 File Supplementary Material. (DOCX)

S2 File PRISMA checklist. (DOC)

## Abbreviations

A-DHAP: alemtuzumab, dexamethasone, cytarabine, cisplatin
AEs: adverse event
ALCL: anaplastic large cell lymphoma
ALCL(+): anaplastic lymphoma kinase positive ALCL
ALCL(-): anaplastic lymphoma kinase negative ALCL
AITL: angioimmunoblastic T-cell lymphoma
auto-HSCT: autologous hematopoietic stem cell transplantation
CHOP: cyclophosphamide, doxorubicin, vincristine, and prednisone
CI: confidence interval
CTCL: cutaneous T-cell lymphoma
DOR: duration of response
ENKTCL: extra-nodal NK/T cell lymphoma, nasal type
ESHAP: etoposide, methylprednisolone, cytarabine, cisplatin
EPOCH: etoposide, prednisolone, vincristine, cyclophosphamide, doxorubicin
FDA: Food and Drug Administration
GDP: gemcitabine, dexamethasone, cisplatin
GemOx: gemcitabine, oxaliplatin
HDAC: histone deacetylase
ICE: ifosfamide, carboplatin, etoposide
MINE: mesna, ifosfamide, mitoxantrone, etoposide
NCCN: National Comprehensive Cancer Network
NHL: non-Hodgkin’s lymphomas
PTCL: peripheral T-cell Lymphoma
PTCL-NOS: peripheral T-cell Lymphoma, not otherwise specified
ORR: overall response rate
OS: overall survival
PFS: progression-free survival
NR: not reported
RCTs: randomized controlled trials
RR: relapsed or refractory
SCT: stem cell transplantation
WHO: World Health Organization
