# Supplementary material for: Highly Diverse Efficacy of Salvage Treatment Regimens for Relapsed or Refractory Peripheral T-Cell Lymphoma: A Systematic Review
Source: PLoS One. 2016 Oct 6;11(10):e0161811. doi: 10.1371/journal.pone.0161811 (PMC5053427; doi:10.1371/journal.pone.0161811)
Supplement: S1 File — (DOCX) [file pone.0161811.s001.docx]

**Supplementary Material 1**

**S1 Table A.** MEDLINE Search Strategy and Results

| Search number | Search Terms | Returns |
| --- | --- | --- |
| #1 | "Lymphoma, T-Cell, Peripheral"[Mesh] | 1186 |
| #2 | "Recurrence"[Mesh] OR "refractory" [Text Word] | 236828 |
| #3 | #1 AND #2 | 132 |
| #4 | "Salvage Therapy"[Mesh] AND "Drug Therapy"[Mesh] | 4604 |
| #5 | "alemtuzumab"[Text Word] OR "belinostat"[Text Word] OR "bortezomib"[Text Word] OR "brentuximab vedotin"[Text Word] OR "cyclosporine"[Text Word] OR "gemcitabine"[Text Word] OR "pralatrexate"[Text Word] OR "romidepsin"[Text Word] OR "DHAP"[Text Word] OR "Dose-adjusted EPOCH"[Text Word] OR "ESHAP"[Text Word] OR "GDP"[Text Word] OR "GemOx"[Text Word] OR "ICE"[Text Word] OR "MINE"[Text Word] OR "carboplatin"[Text Word] OR "cisplatin"[Text Word] OR "cyclophosphamide"[Text Word] OR "cytarabine"[Text Word] OR "dexamethasone"[Text Word] OR "doxorubicin"[Text Word] OR "etoposide"[Text Word] OR "gemcitabine"[Text Word] OR "ifosfamide"[Text Word] OR "mesna"[Text Word] OR "methylprednisolone"[Text Word] OR "mitoxantrone"[Text Word] OR "oxaliplatin "[Text Word] OR "prednisolone"[Text Word] OR "vincristine"[Text Word] | 385804 |
| #6 | #4 OR #5 | 387973 |
| #7 | ("Clinical Trial" [Publication Type] NOT "Clinical Trial, phase I" [Publication Type]) OR "Prospective Studies"[Mesh] | 1047441 |
| #8 | #3 AND #6 AND #7 | 15 |
| #9 | "Comment" [Publication Type] OR "Letter" [Publication Type] OR “Editorial” [Publication Type] OR "Review" [Publication Type] | 3356300 |
| #10 | "Animals"[Mesh] NOT "Humans"[Mesh] | 4009629 |
| #11 | #8 NOT (#9 OR #10) | 14 |

MeSH = Medical Subject Heading (MEDLINE medical index term)

**S1 Table B.** EMBASE Search Strategy and Results

| Search number | Search Terms | Returns |
| --- | --- | --- |
| S1 | 'relapsed peripheral T-cell lymphoma' | 21 |
| S2 | 'refractory peripheral T-cell lymphoma' | 103 |
| S3 | S1 OR S2 | 123 |
| S4 | 'salvage chemotherapy' OR 'alemtuzumab' OR 'belinostat' OR 'bortezomib' OR 'brentuximab vedotin' OR 'cyclosporine' OR 'gemcitabine' OR 'pralatrexate' OR 'romidepsin' OR 'DHAP' OR 'Dose-adjusted EPOCH' OR 'ESHAP' OR 'GDP' OR 'GemOx' OR 'ICE' OR 'MINE' OR 'carboplatin' OR 'cisplatin' OR 'cyclophosphamide' OR 'cytarabine' OR 'dexamethasone' OR 'doxorubicin' OR 'etoposide' OR 'gemcitabine' OR 'ifosfamide' OR 'mesna' OR 'methylprednisolone' OR 'mitoxantrone' OR "oxaliplatin' OR 'prednisolone' OR 'vincristine' | 793,129 |
| S5 | 'Phase II clinical trials' OR 'Prospective study' | [347,496](http://www.embase.com/) |
| S6 | S3 AND S4 AND S5 | 8 |
| S7 | S6 NOT ANIMAL | 8 |
| S8 | S7 AND ([article]/lim OR [article in press]/lim OR [conference paper]/lim) | 4 |

**S1 Table C.** Quality assessment of selected studies (Using Modified 18-item checklist)

|  |  | **Number of Item** | | | | | | | | | | | | | | | | | |  |
| --- | --- | --- | --- | --- | --- | --- | --- | --- | --- | --- | --- | --- | --- | --- | --- | --- | --- | --- | --- | --- |
| **Study (year)** | **Regimen** | **1** | **2** | **3** | **4** | **5** | **6** | **7** | **8** | **9** | **10** | **11** | **12** | **13** | **14** | **15** | **16** | **17** | **18** | **Overall Quality Score** |
| Enblad et al. (2004) | Alemtuzumab | Y | Y | Y | Y | Y | Y | Y | N | Y | Y | Y | U | Y | N | N | Y | Y | N | 13 |
| Foss et al. (2015) | Belinostat | Y | Y | Y | Y | U | Y | Y | N | Y | Y | N | Y | N | N | Y | Y | Y | P | 12 |
| Damaj et al. (2013) | Bendamustine | Y | Y | Y | Y | Y | Y | Y | N | Y | Y | Y | Y | Y | N | Y | Y | Y | Y | 16 |
| Pro et al. (2012) | Brentuximab vedotin | Y | Y | Y | Y | U | Y | Y | N | Y | Y | N | Y | N | Y | Y | Y | Y | P | 13 |
| Horwitz et al. (2014) | Brentuximab vedotin | Y | Y | Y | Y | U | Y | Y | N | Y | Y | N | Y | N | Y | Y | Y | Y | Y | 14 |
| Zinzani et al. (2000) | Gemcitabine | Y | Y | Y | P | Y | Y | Y | N | Y | Y | N | U | U | N | Y | Y | Y | N | 11 |
| Zinzani et al. (2010) | Gemcitabine | Y | Y | N | P | Y | Y | Y | N | Y | Y | N | U | U | N | N | Y | Y | Y | 10 |
| Morschhauser et al. (2013) | Lenalidomide | Y | Y | Y | P | Y | Y | Y | Y | Y | Y | N | Y | U | Y | Y | Y | Y | Y | 14 |
| O'Connor et al. (2011) | Pralatrexate | Y | Y | Y | Y | Y | Y | Y | N | Y | Y | Y | Y | Y | Y | Y | Y | Y | Y | 17 |
| Coiffier et al. (2012) | Romidepsin | Y | Y | Y | Y | U | Y | Y | N | Y | Y | N | P | Y | Y | Y | Y | Y | P | 13 |
| d’Amore et al. (2010) | Zanolimumab | Y | Y | Y | Y | U | Y | Y | U | Y | Y | N | Y | N | N | Y | Y | Y | Y | 13 |
| Huang et al. (2002) | 13-cRA+interferon-α | Y | Y | Y | Y | U | Y | Y | N | Y | Y | N | Y | N | N | Y | Y | Y | P | 12 |
| Seok et al. (2012) | A-DHAP | Y | Y | Y | Y | U | Y | Y | N | Y | Y | N | N | Y | N | Y | Y | Y | P | 12 |
| Zelenetz et al. (2003) | ICE | Y | Y | N | Y | Y | N | Y | N | Y | Y | N | Y | Y | N | Y | N | Y | P | 11 |

Y: yes, N: no, P: partially Reported, U: unclear

The contents of each question are listed as follows:

1. Is the hypothesis/aim/objective of the study clearly stated in the abstract, introduction, or methods section?
2. Are the characteristics of the participants included in the study described?
3. Were the cases collected in more than one centre?
4. Are the eligibility criteria (inclusion and exclusion criteria) to entry the study explicit and appropriate?
5. Were participants recruited consecutively?
6. Did participants enter the study at a similar point in the disease?
7. Was the intervention clearly described in the study?
8. Were additional interventions (co-interventions) clearly reported in the study?
9. Are the outcome measures clearly defined in the introduction or methods section?
10. Were relevant outcomes appropriately measured with objective and/or subjective methods?
11. Were outcomes measured before and after intervention?
12. Were the statistical tests used to assess the relevant outcomes appropriate?
13. Was the length of follow-up reported?
14. Was the loss to follow-up reported?
15. Does the study provide estimates of the random variability in the data analysis of relevant outcomes?
16. Are adverse events reported?
17. Are the conclusions of the study supported by results?
18. Are both competing interest and source of support for the study reported?
